# New Ionic Liquid Microemulsion-Mediated Synthesis of Silver Nanoparticles for Skin Bacterial Infection Treatments

**DOI:** 10.3390/antibiotics12020247

**Published:** 2023-01-25

**Authors:** Fayez Althobaiti, Ola A. Abu Ali, Islam Kamal, Mohammad Y. Alfaifi, Ali A. Shati, Eman Fayad, Serag Eldin I. Elbehairi, Reda F. M. Elshaarawy, W. Abd El-Fattah

**Affiliations:** 1 Department of Biotechnology, Faculty of Sciences, Taif University, P.O. Box 11099, Taif 21944, Saudi Arabia; 2 Department of Chemistry, College of Science, Taif University, P.O. Box 11099, Taif 21944, Saudi Arabia; 3 Department of Pharmaceutics, Faculty of Pharmacy, Port Said University, Port Said 42526, Egypt; 4 Biology Department, Faculty of Science, King Khalid University, Abha 62529, Saudi Arabia; 5 Cell Culture Lab, Egyptian Organization for Biological Products and Vaccines (VACSERA Holding Company), 51 Wezaret El-Zeraa St., Agouza, Giza 12654, Egypt; 6 Department of Chemistry, Faculty of Science, Suez University, Suez 43533, Egypt; 7 Institut für Anorganische Chemie und Strukturchemie, Heinrich-Heine Universität Düsseldorf, 40204 Düsseldorf, Germany; 8 Chemistry Department, College of Science, IMSIU (Imam Mohammad Ibn Saud Islamic University), P.O. Box 5701, Riyadh 11432, Saudi Arabia; 9 Department of Chemistry, Faculty of Science, Port Said University, Port Said 42526, Egypt

**Keywords:** benzyl alkyl imidazolium ionic liquids, microemulsions, silver nanoparticles, antibacterial, skin bacterial infection treatments

## Abstract

This work reports a new approach for the synthesis of extremely small monodispersed silver nanoparticles (AgNPs) (2.9–1.5) by reduction of silver nitrate in a new series of benzyl alkyl imidazolium ionic liquids (BAIILs)-based microemulsions (3a–f) as media and stabilizing agents. Interestingly, AgNPs isolated from the IILMEs bearing the bulkiest substituents (*tert*-butyl and *n*-butyl) (3f) displayed almost no nanoparticle agglomeration. In an in vitro antibacterial test against ESKAPE pathogens, all AgNPs-BAIILs had potent antibiotic activity, as reflected by antibacterial efficiency indices. Furthermore, when compared to other nanoparticles, these were the most effective in preventing biofilm formation by the tested bacterial strains. Moreover, the MTT assay was used to determine the cytotoxicity of novel AgNPs-BAIILs on healthy human skin fibroblast (HSF) cell lines. The MTT assay revealed that novel AgNPs-BAIILs showed no significant toxic effects on the healthy cells. Thus, the novel AgNPs-BAIILs microemulsions could be used as safe antibiotics for skin bacterial infection treatments. AgNPs isolated from BAIIL (3c) was found to be the most effective antibiotic of the nanoparticles examined.

## 1. Introduction

The exponential increase in bacterial-induced pathogenic infection has created a serious threat to global human health. The ESKAPE pathogens (*Staphylococcus aureus* (*SA*), *Klebsiella pneumoniae* (*KP*), *Pseudomonas aeruginosa* (*PA*), *Enterococcus faecium* (*EF*), Acinetobacter baumannii (*AB*), and Enterobacter species) have been assigned as the most deadly bacteria by the Infectious Diseases Society of America (IDSA). Antimicrobial agents (AMAs) effectively protect humans from these potentially fatal pathogenic micro-organisms [1,2]. However, many bacterial strains acquired tolerance to antibiotics long before humans began mass-producing them to prevent and cure infectious illnesses [3]. Further, the emergence of antimicrobial-resistant microbes has sparked a worldwide crisis due to antibiotic overuse and the limited effectiveness of conventional antibiotic therapy. Consequently, research for novel antimicrobial agents is urgently required to address this issue.

Recently, nanomaterials (NMs) have attracted a lot of attention from scientists and found widespread usage in many biological applications [4,5,6,7]. Among the different nanomaterials, noble metal nanoparticles (NMNPs) have gained great attention in the development of a diversity of smart multifunctional NMs for biomedical applications, owing to their non-toxicity and excellent intrinsic properties [8]. Aside from being surface active, NMNPs can release bioactive metal ions into biological systems, resulting in the induction of multiple modes of bioactivity.

There has been extensive research into nanosystems for drug delivery because of its attractive biodegradability, biocompatibility, specificity/selectivity, and low toxicity. In addition to its useful properties including biocompatibility and durability, non-immunogenicity, a large surface area, a high drug loading capacity, and a minimal leakage of medications, they can also be employed to the targeted administration of pharmaceuticals [9]. Over the past two decades, the use of metal nanoparticles (MNPs) as nanocarriers has drastically evolved due to their numerous advantages and benefits. Their unique physical, chemical, and biological properties are in the forefront of these advantages. Additionally, their small size allows them to traverse through biological barriers and release drugs at a desired target site [10]. Moreover, MNPs possess high loading capacity, surface-area-to-volume ratio, and can be easily functionalized with a variety of ligands to modulate the drug release profile. Therefore, MNPs have been successfully used in a range of drug delivery systems, including transdermal, oral, and injectable delivery [11]. Despite the advantages, the use of MNPs as nanocarriers is still in its infancy due to several current challenges. For example, the toxicity of MNPs, the difficulty in controlling their size and shape, and poor biocompatibility are several factors that need to be addressed [10].

Despite several studies reporting on the biological and therapeutic applications of NMNPs, particularly AgNPs [12,13,14], there have been very few reports on the use of ionic liquid-supported AgNPs (PdNPs-ILs) in these disciplines. For instance, Dorjnamjin et al. reported the synthesis of uniform monodisperse crystalline Ag nanoparticles mediated by two different series of hydroxyl functionalized ionic liquids (HFILs) and hydroxyl functionalized cationic surfactants (HFCSs). AgNPs isolated from various ionic liquids exhibited promising in vitro antimicrobial activities against a range of Gram-positive and Gram-negative bacteria and fungi [15]. In addition, a room temperature ionic liquid (2-amino-1-dodecylpyridinium bromide) was used to prepare AgNPs (2–20 nm) with excellent antibacterial activity against *S. aureus, E. coli, and P. aeruginosa* [16]. AgNPs anchored in poly(ionic liquid) mesoporous nanocomposite (Ag-PIL) were recently synthesized by in situ reduction of AgNO_3_ in PIL and used for controlled anticancer drug delivery with an antimicrobial effect. The Ag-PIL nanocomposite demonstrated outstanding bacteriostatic and bactericidal activity against both *E. coli* and *S. aureus* [17].

Notably, one of the drawbacks of using MNPs is their proclivity to agglomerate and aggregate as a result of the Ostwald ripening process [18]. This significantly reduces their stability and limits their utility in pharmaceutical applications [19]. Therefore, the MNPs should be stabilized either sterically or electrostatically to prevent agglomeration [19]. Benzyl alkyl ionic liquids (BAILs) could offer a promising solution for steric and electrostatic stabilizing of MNPs [20]. Electrostatic and steric interactions between MNPs and ILs contribute to their stabilization without affecting surface characteristics [21,22]. Furthermore, the ILs’ strong ionic strength, polarity, and dielectric constant make them suitable mediums for the preparation and stabilization of MNPs [22]. On the other hand, among the various reported methods for synthesizing NMNPs [23], the microemulsion approach has attracted the attention of many researchers worldwide due to its simplicity, cost-effectiveness, and efficiency to produce stable NPs [24,25].

Motivated by these astounding facts and as a new step in our ongoing journey to explore and develop novel pharmacological agents [26,27,28], the present study reports the synthesis of new BAIILs for application in the preparation and stabilization of AgNPs. In this study, several BAIIL aggregation effects will be investigated as a function of the phenyl and imidazyl substituents. Furthermore, the effects of various produced AgNPs on ESKAPE pathogens as well as healthy human skin fibroblast (HSF) cell lines will be examined.

## 2. Results and Discussion

### 2.1. Synthesis

**Figure 1:**
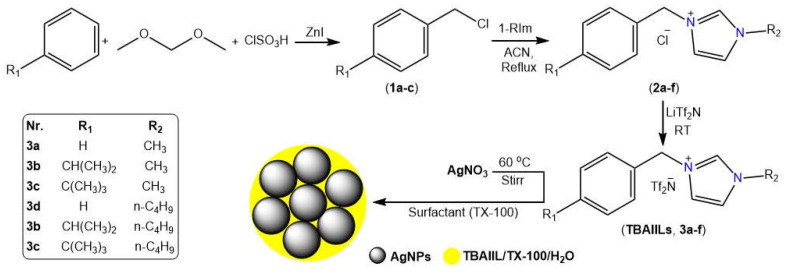
Stepwise synthesis of BAIILs (**3a–f**) their applications in the synthesis of AgNPs.

Using substituted alkylbenzenes and alkyl imidazoles as building blocks, a three-step methodology is developed to produce the desired benzyl alkyl ionic liquids (BAIILs) (See Figure 1). First, alkylbenzenes (cumene and tert-butylbenzene) were chloromethylated with a chloromethylating agent mixture of dimethoxymethane-chlorosulfonic acid-ZnI to produce the appropriate chlorobenzyl derivatives (**1a–c**). Following that, N-benzyl imidazolium chlorides (**2a–f**) were synthesised by quaternizing 1-alkylimidazoles with chlorobenzyl derivatives under refluxing conditions in an inert atmosphere. Eventually, the counterparts imidazolium bis-((trifluoromethyl)sulfonyl) imide ionic liquids (**3a–f**) were obtained by subjecting these ILs to anion (chloride) metathesis with LiTf_2_N at room temperature. On the other hand, the AgNPs were successfully produced using hydrazine hydrate-catalyzed AgNO_3_ reduction in BAIILs/TX-100/H_2_O microemulsions. The colour change of water-BAIIL microemulsion containing Ag+ ions from a light yellow to a brownish yellow is indicative of the AgNPs formation. In this reduction process, BAIIL acts as a green solvent and stabilizing agent (Figure 1).

### 2.2. Physical Characterization

BAIILs were produced in excellent yields (85–95%) overall and they were physically characterized based on their appearance, solubility, lipophilicity, viscosity, and thermal stability measurements.

#### 2.2.1. Physical Appearance, Solubility, and Lipophilicity

It is well established that the aqueous solubility and lipophilic properties of a new pharmacological agent are directly related to its pharmacokinetics and pharmacodynamics. It was for this reason that the room-temperature aqueous solubility of the novel BAIILs was studied. All BAIILs were found to be soluble in water, though each BAIIL dissolved in different degree depending on its structure. The degree to which they are soluble in water is controlled by the type of alkyl substituent present on the benzene and imidazole rings (see Table 1). For example, the tert-butylbenzylimidazolium cation (**3f**), which bears the most hydrophobic side chain (n-butyl), is the least soluble of the group (LogS = −8.163), while the benzylmethylimidazolium cation (**3a**) has the highest solubility (LogS = −5.719).

CLogP measurements demonstrate that the benzyl-alkylimidazolium cation is the least lipophilic cation, with a range from (−0.352) to (−0.102) depending on the nature of the alkyl substituent used (see Table 1). In contrast, replacing the hydrogen atom on the benzene ring with more hydrophobic groups such as *iso*-propyl and *tert*-butyl has significantly increased the CLogP values to be in the range of (−0.262)–(1.325) and (0.137)–(1.724), respectively, confirming their great lipophilic character. Interestingly, ionic liquids interact strongly with the outer lipophilic layer of microbial cell surfaces when they have a high CLogP value, and consequently their lipophilicity is increased [29].

#### 2.2.2. Viscosity and Thermal Stability

Notably, IL-viscosity exhibits a great influence on the formation, molecular diffusion, and stability of nanoparticles. The stability of nanoparticles is greatly enhanced by the fact that their diffusion is greatly reduced in extremely viscous media like ionic liquids, which results in a lifetime increase of a factor of 10–1000 compared to that in traditional low viscosity solvents [30]. Ionic liquids also have the added benefit of reducing the likelihood of agglomeration of colloidal nanoparticles by suppressing their thermal motion due to the high viscosity. Therefore, the viscosities of new BAIILs were measured at 25 °C, and the results are shown in Table 1. High viscosity values (421.15–543.25 cP) were observed for all imidazolium ILs; however, these values varied according to the cation’s intrinsic structural characteristics. For example, out of all of the examined ionic liquids, the one with the lowest viscosity (428.75 cP) was BAIIL **3a**, which was made up of the simplest cation (benzyl-methylimidazolium). In contrast, tert-butylbenzyl group-containing BAIIL **3f** showed the greatest viscosity (543.25 cP). The very hydrophobic Tf_2_N anion exerts stronger ion–ion interactions with the hydrophobic tert-butylbenzyl-methylimidazolium cation, which leads to the observed behavior of an increase in viscosity [29].

On the other hand, the thermal stabilities of BAIILs (**3a–f**) were verified using their thermogravimetric (TG) curves (Figure S1, Supplementary Materials). All BAIILs are clearly thermally stable up to about 400 °C before undergoing a sudden decline in their masses between 400 and 450 °C. Table 1 and Figure S1 (Supplementary Materials) show that BAIILs with long side chains (n-butyl) linked to the imidazolium ring (**3e–f**) had more complex thermal degradation patterns and lower decomposition temperatures than BAIILs containing methylimidazolium cation (**3a–c**).

### 2.3. Structural Characterization

Spectral investigations (FTIR, NMR (^1^H, ^13^C, ^19^F), and ESI-MS) were used to deduce the structural formulae of all the synthesized BAIILs.

#### 2.3.1. Mass Spectrometry

**Figure 2:**
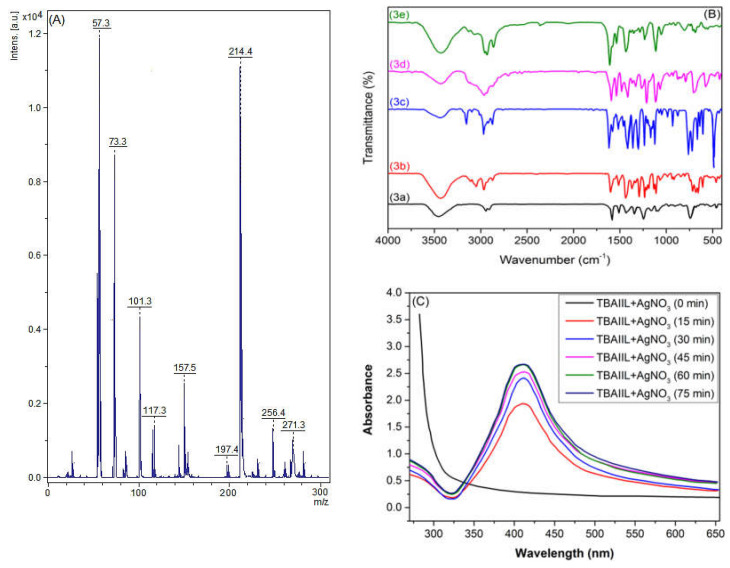
(**A**) A positive mode electrospray ionization mass spectra (ESI-MS (+ve)) of BAIIL (**3f**); (**B**) FTIR spectra of the native BAIILs (**3a–f**); and (**C**) UV-Vis spectrum AgNPs produced by BAIIL (**3f**).

To acquire a first perception of the characteristics of their cation and anion’ structures, the electrospray ionization mass spectra (ESI-MS) of BAIILs can be a helpful tool. In light of this, the ESI-MS of BAIIL (**3f**) (Figure 2A) was extensively studied as a representative of new BAIILs. The major peak was found at an *m*/*z* value of 271.3, which corresponds to the molar mass of a single-charged cation, [M − Tf_2_N^−^]^+^, generated by the elimination of the bonded anion. In addition, the fragmentation peaks that can be seen at *m*/*z* 214.4, 157.5, and 57.3 (base peak) could be assigned to consecutive removal of butyl side chains and benzyl-butylimidazolium radicals from the parent molecule, respectively, [M − Tf_2_N^−^ − C_4_H_9_**˙**]^+^, [M − Tf_2_N^−^ − 2 (C_4_H_9_**˙**)]^+^, and [M − Tf_2_N^−^ − BnBIm**˙**]^+^.

#### 2.3.2. FTIR Spectroscopy

The FTIR spectra of the new BAIILs (Figure 2B) were studied in an effort to learn more about the structural features of the cations and anions that make them up. The spectra of the TAILs exhibit absorption bands that are analogous to those of previously reported imidazolium-based ILs [31,32,33]. The common absorption peaks that can be seen in the FTIR spectra of BAIILs at 3110, 2970, 1591, 1245, 875, and 707 cm^−1^ could be attributed to the vibrational modes of the benzylimidazolium cation fragments, including imidazolium C2-H, alkyl C-H, imidazolium C=N, and the benzyl moiety, respectively [31]. In addition, the distinctive vibration bands of (TN_2_f) anion may be observed in the regions of 1271 ± 3 cm^−1^ ascribed to *ν*_as_(CF_3_ + SO_2_); 1224 ± 2 cm^−1^ due to *ν*_s_(CF_3_ + SO_2_); 1139 ± 4 cm^−1^ typical for *ν*_as_(CF_3_ + CS); 1024 ± 3 and 911 ± 3 cm^−1^ for *ν*(N-S); 710 ± 5 cm^−1^ for δ(CF_3_); and 747 ± 3 cm^−1^ assigned to *ν*(C-S) [34].

#### 2.3.3. UV-Vis Spectroscopy

Figure 2C shows UV-Visible spectra of the BAIIL (**3f**)-based microemulsion containing AgNO_3_ at time intervals as well as the progress in the formation of BAIIL-stabilized AgNPs with time. The appearance of a new peak at 430 nm in the microemulsion spectrum, which is characteristic of the surface plasmon resonance (SPR) of AgNPs [35], verifies the synthesis of AgNPs. Further, the SPR peak intensity rises with time, reflecting an increase in AgNPs yield, until it reaches a maximum value at 60 min. Thereafter, the peak intensity almost stops rising, denoting the end of the reaction.

#### 2.3.4. NMR Spectroscopy

**Figure 3:**
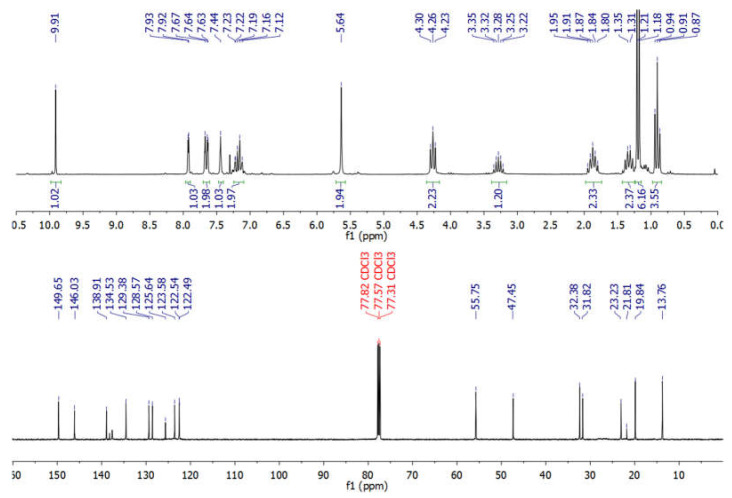
^1^HNMR (200 MHz) and ^13^CNMR (125 MHz) of BAIIL (**3e**) in CDCl_3_.

NMR spectra of new BAIILs were utilized to verify their successful production and provide a striking visual representation of the structure of their ions. However, due to the fact that all new BAIILs have nearly identical NMR spectra, with the exception of the peak of alkyl side chains, the ^1^H/^13^C NMR spectra of BAIIL (**3e**) (Figure 3) were analyzed in more detail as a representative of new BAIILs. As shown in the ^1^H NMR spectrum of **3e**, the proton resonance of imidazolium (C2-H) can be seen as a singlet at 9.91 ppm. Furthermore, a group of signals was detected in the chemical shift region of 7.93–7.12 ppm, assignable to the resonances of the imidazolium and phenyl protons. In addition, the benzylic protons can be seen as a singlet peak at 5.64. As for the protons of alkyl side chains (isopropyl and n-butyl groups), the methine and methyl protons of the isopropyl group emerged as septet (3.28 ppm) and doublet (1.21 ppm), respectively. While the methylene and methyl protons of the n-butyl group can be observed as a set of multiplets in the high-field region (4.26–0.91 ppm). The ^13^C NMR spectrum of **3e**, on the other hand, shows the carbon map in detail for both the central benzylimidazolium cation and the alkyl substituents. The carbon signals characteristic of the benzylimidazolium cation can be detected in the low-field region (146.03–122.54 ppm) for the resonances of the imidazolium and phenyl carbon atoms, while it is 55.75 for benzylic carbon. In contrast, the peaks distinctive of C-atom of alkyl substituents can be seen in the high-field region, 32.38 and 23.23 ppm for methine and methyl carbons of the isopropyl group; and 47.45, 31.82, 19.84, and 13.76 ppm for carbon atoms of n-butyl group. It is worth noting that the low-field carbon signal observed at 149.65 could be assigned to CF_3_ of Tf_2_N anion [29].

### 2.4. Morphological Characterization

#### 2.4.1. Transmission Electron Microscopy (TEM) Analysis

**Figure 4:**
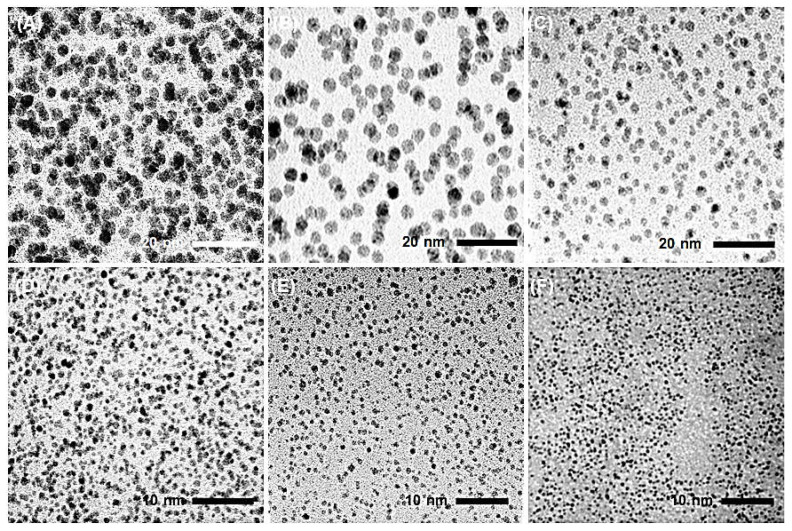
TEM images of AgNPs generated by the hydrazine-reduction of AgNO_3_ in different BAIILs-based microemulsions: (**A**) 3a, (**B**) 3b, (**C**) 3c, (**D**) 3d, (**E**) 3e, and (**F**) 3f.

Representative TEM images of AgNPs obtained from hydrazine reduction of AgNO_3_ in various BAIILs are shown in Figure 4. When AgNO_3_ is reduced in the presence of new BAIILs, discrete AgNPs with diameters in the range of 2.9–1.5 nm are formed (from TEM). More agglomeration can be seen in the AgNPs made from the methylimidazolium-supported BAIILs (3a–c) (Figure 4A–C) than in those made from the butylimidazolium-supported BAIILs (**3d–f**) (Figure 4D–F). Meanwhile, the primary AgNPs produced by 4-iso-propylbenzyl- or 4-tert-butylbenzyl-substituted BAIILs (**3b**, **3c**, **3e**, and **3f**) were more separated than those produced by unsubstituted benzyl-based BAIILs (**3a**,**d**). This could be due to the electrostatic and steric interactions between AgNPs and BAIILs, which contribute to their stabilization without affecting surface characteristics by forming a protecting layer that prevents AgNPs coalescence [21,22]. Unlike more traditional approaches, this synthetic process permits the synthesis of AgNPs networks with narrower particle size dispersion. Interestingly, AgNPs isolated from the BAIIL bearing the bulkiest substituents (*tert*-butyl and *n*-butyl) (**3f**) displayed almost no NPs agglomeration (see Figure 4E).

#### 2.4.2. Particle Size Distribution (PSD)

**Figure 5:**
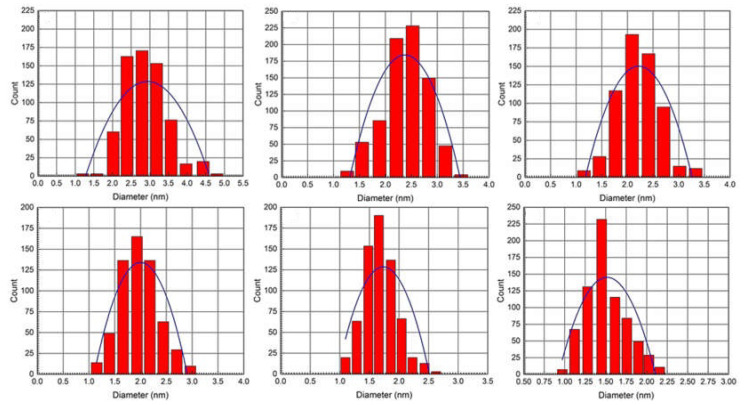
PSD histograms of AgNPs obtained using different BAIILs-based microemulsions.

The size histograms of the AgNPs obtained by BAIILs (Figure 5) show that the nanoparticles have a very narrow PSD and are either not agglomerated or exhibit very little agglomeration. Uniformly dispersed AgNPs with a mean diameter of 1.5 ± 0.5 nm were produced by using the BAIIL **3f** with the highest surface steric energy (69.772 kcal/mol) as a stablizing agent. In contrast, when using BAIIL **3a** of lowest surface steric energy (50.029 kcal/mol) as a medium for AgNPs production, the AgNPs with a mean diameter of 2.9 ± 0.6 nm were obtained coupled with few agglomerated big AgNPs cluster of 4–5 nm.

### 2.5. Antibacterial Assay

**Figure 6:**
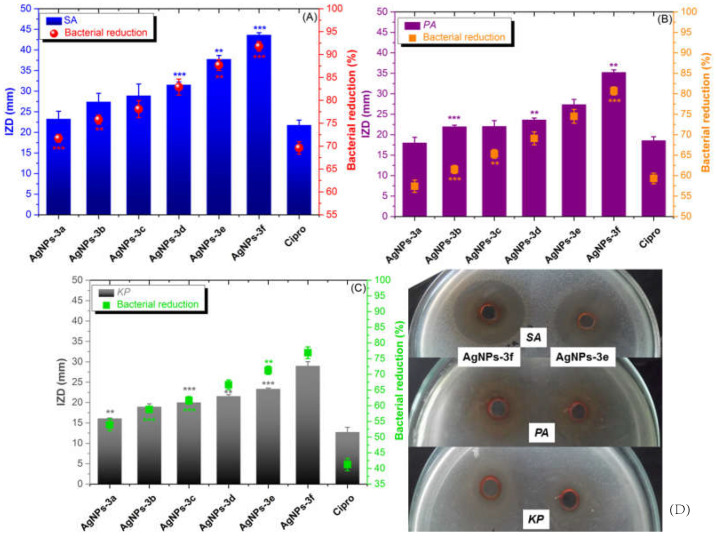
Graph for the inhibition zone diameter (IZD, mm) and the percentage of bacterial colonies reduction (%) for the examined AgNPs against (**A**) G^+^ bacteria (*SA*), (**B**) G^−^ bacteria (*PA*), and (**C**) G^−^ bacteria (*KP*) (** *p* < 0.005, *** *p* < 0.001). (**D**) Photographs of inhibition zones for the most active antibiotics (**AgNPs-3e** and **AgNPs-3f**).

The in vitro antibacterial activity of BAIILs-coated AgNPs was evaluated in comparison to that of Ciprofloxacin (Cipro) (a clinical antibiotic used for treating skin bacterial infections) using three of the most common ESKAPE infections found in contaminated food. Initially, the antibacterial efficacy of each sample was measured using the inhibition zone diameter (IZD, mm). As can be seen in Figure 6, all AgNPs have the capacity to limit the growth of all tested bacterial cells; however, their efficacy varies depending on the kind of bacterium, BAIIL structural characteristics, and AgNPs’ mean size. Noteworthy, the gram-positive (G^+^) bacterial strain (*SA*) was generally more susceptible to all treatments than gram-negative (G^−^) ones (*PA* and *KP*). The structural differences between the outer bacterial walls of the two types may be to blame for the different inclination of bacterial membrane permeability and, as a result, bactericidal effects. Particularly, unlike the G^+^—bacterial wall that contains only a thin peptidoglycan layer, G^−^- bacteria have a more sophisticated outer membrane that may operate as a barrier to the invasion of antibiotics into bacterial cells due to the presence of phospholipids (PLs), lipopolysaccharides (LPS), and lipoproteins (LPs) [36]. Interestingly, the IZD data indicate that the antibacterial activity of AgNPs improves as their mean size falls. For example, the AgNPs of the smallest size (1.5 nm), obtained using BAIIL **3f**, exhibit the highest activity against *SA* (43.59 ± 1.48 mm). In contrast, the antistaphylococcal activity (24.31 ± 0.79 mm) is lowest for the biggest AgNPs (2.9 nm), which were made using BAIIL **3a**. The findings of CFU method and CFU/mL values are in good consistency with the results obtained by AWD method. As shown in Figure 6A–C and Figure S2 (SM†), a remarkable bacterial reduction was observed in all bacterial cells after treatment with BAIIL-coated AgNPs; however, the performance depends on the bacterial strain type, the ionic liquid coating, and the AgNPs’ sizes. Overall, the G^+^ strain (*SA*) was more sensitive to AgNPs, and its bacterial colony count was reduced by a value of 72–92% after treatment. In contrast, G^−^ bacteria (*PA* and *KP*) were less responsive to AgNPs treatments and showed bacterial colony reductions (BCR) of 58–81% and 54–75%, respectively, in AgNPs-treated PA and KP samples in comparison to growth controls.

It is worth noting that the butylimidazolium-coated AgNPs (AgNPs-3d, AgNPs-3e, and AgNPs-3f) (BCR 68–92%) are more potent antibiotics than methylimidazolium-coated AgNPs (AgNPs-3a, AgNPs-3b, and AgNPs-3c) (BCR 54–77%). Antibiotic activity (BCR 71–92%) is greatest for AgNPs with a mean particle size of 1.5 nm.

Once more, Table 2 MIC and MBC values demonstrate that G^+^-bacterium was more sensitive to AgNPs than G^−^ species. The presence of negatively charged phosphate groups on its surface may also play a role in this [37]. The positively charged nanoparticles can interact strongly with these groups. The extent to which AgNPs exhibited bactericidal or bacteriostatic actions was also significantly influenced by the type of tested bacteria and the NPs size. For example, the AgNPs coated by 4-tert-butylbenzyl-substituted BAIIL (**3f**) were the most potent antibiotic for *SA* (MIC/MCB = 0.25 ± 0.12/0.35 ± 0.16 μg/mL).

On the other hand, AgNPs obtained by nascent benzyl-imidazolium BAIIL (**3a**) were the least active anti-staphylococcal agent (MIC/MCB = 3.25 ± 0.25/3.75 ± 0.31 μg/mL). It is worth noting that with MIC/MCB values between 2.22 ± 0.15/2.25 ± 0.19 μg/mL and 9.32 ± 0.34/9.50 ± 0.37 μg/mL, *KP* is the most drug-resistant strain of bacteria.

### 2.6. Anti-Biofilm Activity

**Figure 7:**
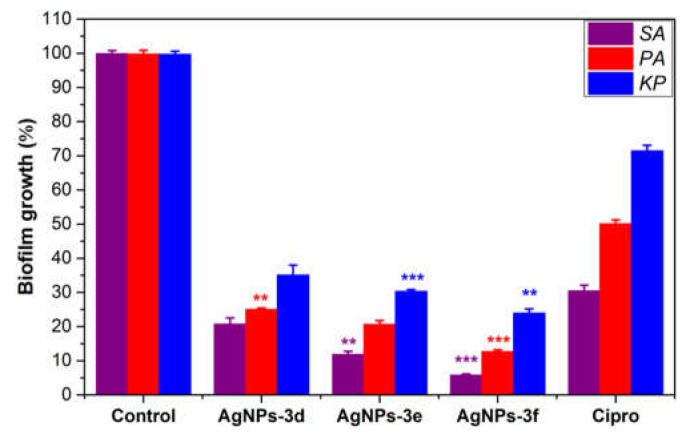
Inhibition of bacterial biofilm formation by the most potent antibiotics (AgNPs-3d, AgNPs-3e, and AgNPs-3f) as compared to a positive control (Cipro) and negative control (DI water), (** *p* < 0.005, *** *p* < 0.001).

The ability of the most potent antibiotics (**AgNPs-3d**, **AgNPs-3e**, and **AgNPs-3f**) to prevent the development of bacterial biofilm on polystyrene surfaces was evaluated in vitro as compared to a positive control (Cipro) and a growth control (deionized water, DIW). As can be seen in Figure 7, all of the materials studied strongly limit the formation of bacterial biofilms, albeit this capacity varies depending on material structure and bacterial type. Specifically, the G^+^ bacterial biofilm (*staphylococcal* biofilm) formation is inhibited by AgNPs more so than by G^−^ bacterial (*PA* and *KP*) biofilms. Furthermore, it is evident that AgNPs impeded *PA* biofilm formation more so than *KP* biofilm production (*p* < 0.005). Among the tested AgNPs, (tert-butyl)benzyl)-butylimidazolium-coated AgNPs (**AgNPs-3f**) was the most effective anti-biofilm agent, inhibiting bacterial biofilm formation by approximately 96%, 89%, and 78% for *SA*, *PA*, and *KP*, respectively, which was 1.5- to 2-fold higher than the effects induced by the positive control (Cipro). These findings suggest that the increased activity of AgNPs in preventing bacterial biofilm formation is due to its strong antimicrobial impact on the bacterial cells submerged in cultures or biofilms, and their ability to restrict adhesion of bacterial cells onto the NPs-coated polystyrene surfaces.

### 2.7. In Vitro Cytotoxicity

**Figure 8:**
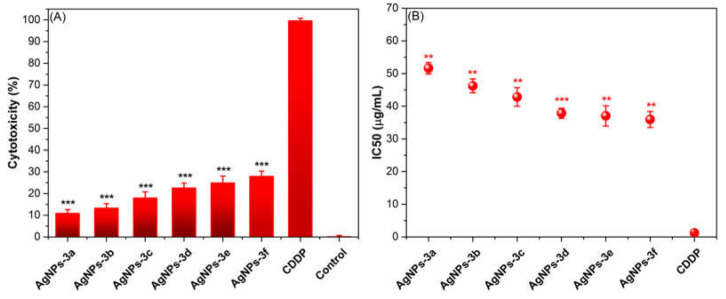
(**A**) A single dose (10 μg/mL) inhibitory impacts of BAIILs-AgNPs on the proliferation of HSF cell lines. (**B**) Values of IC_50_ (μg/mL) for BAIILs-AgNPs against HSF in comparison to CDDP (** *p* < 0.005, *** *p* < 0.001).

New BAIILs-coated AgNPs were tested for cytotoxicity against normal (HSF) cells using the MTT assay in comparison to the positive control, cisplatin (CDDP). It is common practice to conduct initial single-dose studies of novel drugs for cytotoxic effects in human cell lines. Therefore, we looked at how BAIILs-coated AgNPs affected HSF cell proliferation when used in a single dose (10 μg/mL) (Figure 8A). The cytotoxicity data showed that all BAIILs-coated AgNPs are significantly (*p* < 0.0001) less toxic than CDDP toward HSF cells. In addition, the AgNPs derived from the butylimidazolium-supported BAIILs (**3d–f**) (AgNPs-3d, AgNPs-3e, and AgNPs-3f) are more toxic for HSF cells than the AgNPs obtained from the methylimidazolium-supported BAIILs (**3a–c**). Meanwhile, according to IC_50_ values (Figure 8B), the clinical drug (CDDP) is more toxic to healthy cells than all new TBAIILs-AgNPs. BAIIL coatings are proving to be an effective tool in reducing the toxic effects of silver nanoparticles on normal cells. This new innovative solution is based on using BAIILs covalently bound to the silver nanoparticles. These ILs act to reduce the release of silver ions, which are typically the most toxic components. According to our findings, this study provides hope for the future development of safe and promising BAIILs-AgNPs-based microemulsions as bacterial infection medications, particularly for skin bacterial infections.

### 2.8. Proposed Mechanism for Pharmacological Activity of New BAIILs-AgNPs

**Figure 9:**
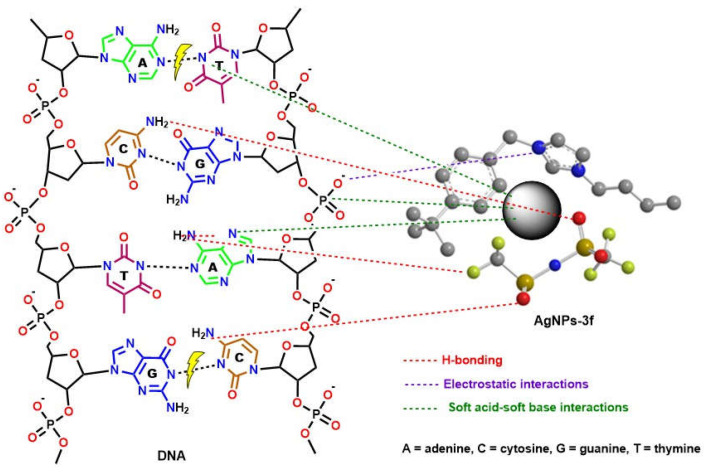
The proposed mechanism for DNA-cleavage activity of new thiazolium AgNPs-3f.

There is still much uncertainty about how NMNPs exert their beneficial effects on bacteria or cancer. However, the high biocompatibility and excellent photothermal effects of AgNPs may greatly contribute to their superior bioactivity [38]. In addition, the ability of BAIIL-coated AgNPs to adhere to the bacterial membrane by electrostatic binding between the negatively charged bacterial cell and the positively charged NPs and BAIIL is critical for their bactericidal activity. This breaks the integrity of the bacterial membrane, leading to cell death [39]. Moreover, the extremely small sizes (2.9–1.5 nm) and unique hydrophobic coatings (**BAIILs**) enable these NPs to enter bacterial cells without being ingested by endocytosis and to subsequently aggregate within the cells, where they can exert a wide range of antimicrobial effects [39,40]. According to Jiang et al., silver nanoparticles release silver ions that interact with the thiol groups of many enzymes, rendering most of the respiratory chain enzymes inactive and thereby triggering the formation of reactive oxygen species (ROS), which in turn triggers the bacterial cell’s own self-destruction and that of the cancer cell as well [41]. Additionally, silver works as a soft acid that interacts readily with the nitrogen, sulphur, and phosphorus bases of DNA to inactivate its replication, so rendering the nuclear machinery of the cell inoperable [42]. Eventually, it could be speculated that the surface area to volume ratio of AgNPs has a significant impact in providing pharmacological activity. The presence of BAIILs capping nanoparticles confers a unique surface functionality, causing them to interact with various cell types in a predetermined fashion (see Figure 9). The effectiveness of pharmacological activity increases as particle size decreases. Additionally, the BAIIL coating plays an important role in the enhancing the antibacterial action of AgNPs in multiple possible ways: (i) the effects on bacterial cell walls due to interactions between cationic imidazolium group and their negative charge; (ii) the capabilities of hydrophobic alkyl substituents to aid AgNPs in penetrating lipophilic cell membranes [43]; and (iii) changes in membrane structure and dynamics as a result of exposure to imidazolium-based ionic liquids. Specifically, the imidazolium-based ionic liquids caused changes in the lipid bilayer of the cell membrane, leading to an increase in membrane permeability and cellular damage [44].

Comparing the antibacterial activity of newly developed BAIILs-capped AgNPs with that of previously reported counterparts (see Table S1, Supplementary Materials), [45,46] revealed that the BAIILs-AgNPs had significantly higher antibacterial efficacies (with MIC/MBC values in the range of 0.25/0.35–2.22/2.25 μg/mL) than the previously reported AgNPs (with MIC/MBC values in the range of 16/16–256/256 μg/mL). These results demonstrate that the new AgNPs have both higher MIC values as well as higher MBC values in comparison to previously reported ionic liquid-coated AgNPs. This indicates that the new AgNPs are more effective in controlling bacteria growth than their previously studied counterparts. Thus, the new AgNPs could potentially be an effective and safe alternative to treat bacterial infections more effectively.

Notably, the alkyl chain length of BAIILs plays vital roles both in the steric stabilization of AgNPs as well as their antibacterial capabilities. The findings of the previous studies indicate that increasing the chain length of the ionic liquid can enhance the steric stabilization of AgNPs, which can be attributed to the increased number of hydrophobic interactions between the ionic liquid molecules and the AgNPs [45,46]. In addition, increasing the alkyl chain length of an imidazolium ionic liquid can lead to an increase in its antimicrobial effectiveness. The findings are in agreement with a study conducted by Docherty and Kulpa [47] which revealed that chain length has a significant impact on the antimicrobial potency of the ionic liquids. The researchers found that increasing the chain length from C1 to C4 resulted in a significant increase in antibacterial activity against Gram-positive and Gram-negative bacteria. In particular, the C4 derivative was observed to be the most effective with a minimum inhibitory concentration (MIC) of 8.25 mM against G^+^-bacteria and 4.03 mM against G^−^-bacteria. The increased antibacterial activity was due to an increased partition coefficient of the ionic liquid, which allowed it to be more effectively absorbed by the cellular membrane of the bacteria.

## 3. Materials and Methods

Chemical and solvent suppliers and their details were provided in the Supplementary Materials (Supplementary Materials). In addition, the preparation and characterization of benzyl chloride (R^1^BnCl) derivatives (**1a–c**) and benzyl alkyl imidazolium chloride [R^1^BnImR^2^]^+^Cl^−^ ionic liquids (**2a–f**) were described in the Supplementary Materials.

The new BAIILs were structuraly chracterized based upon the spectral analyses (FTIR, UV-Vis, NMR (^1^HNMR, ^13^CNMR, ^19^FNMR), and ESI-MS) and physical measurements. The detail for these instruments were also provided in the Supplementary Materials.

### 3.1. Synthesis of Tunable Benzyl Alkyl Imidazolium Ionic Liquids (BAIILs, 3a–f)

While vigorously stirring, a solution of lithium bis(trifluoromethanesulfonimide) (LiTf_2_N) (6.66 g, 0.03 mol) in a combination of ACN (10 mL) and deionized water (DIW) (10 mL) was added dropwise to a solution of [R^1^BzR^2^Im]Cl (**2a–f**) (0.03 mol) in DIW. The resulting mixture was then magnetically stirred overnight at room temperature. After the reaction time was completed and the aqueous layer was discarded, the oily residues were dissolved in dichloromethane (DCM) and repeatedly washed with DIW until there was no precipitation of the AgNO_3_ solution with the washing water. The resulting oily products were subsequently vacuum-dried for 48 hours at 343 K to remove any leftover water. Samples of the produced BAIILs (**3a–f**) were chracterized as follow:

**3-benzyl-1-methylimidazolium bis((trifluoromethyl)sulfonyl)amide** [BnMIm][Tf_2_N] (**3a**): Obtained in a 95% yield. ^1^H NMR (500 MHz, CDCl_3_) δ (ppm); 9.41 (1H, s, Im-H), 7.90 (1H, d, J = 1.8 Hz, Im-H), 7.72 (1H, d, J = 1.8 Hz, Im-H), 7.70–7.49 (5H, m, Ar-H), 5.38 (2H, 2, Ph-CH_2_), 3.87 (3H, s, N-CH_3_). ^13^C NMR (75 MHz, CDCl_3_) δ (ppm): 149.87, 139.96, 137.38, 131.42, 129.56, 126.21, 124.51, 122.98, 56.39, and 37.63. ^19^F NMR (565 MHz, CDCl_3_): singlet at δ −81.67 ppm (Tf_2_N-CF_3_). ESI-MS (positive mode): 173.2 *m*/*z* [M − Tf_2_N^−^, C_11_H_13_N_2_]^+^.

**3-(4-isopropylbenzyl)-1-methylimidazolium bis((trifluoromethyl)sulfonyl)amide** [*^iso^*PBnMIm][Tf_2_N] (**3b**): Obtained in a 87% yield. ^1^H NMR (500 MHz, CDCl_3_) *δ* (ppm); 9.35 (1H, s, Im-H), 7.96 (1H, d, *J* = 1.9 Hz, Im-H), 7.74 (1H, d, *J* = 1.9 Hz, Im-H), 7.38–7.16 (4H, m, Ar-H), 5.39 (2H, 2, Ar-CH_2_), 3.98 (3H, s, N-CH_3_), 3.78 (1H, p, *J* = 1.9 Hz, CH(CH_3_)_2_), 1.24 (6H, d, *J* = 6.9 Hz, CH(CH_3_)_2_). ^13^C NMR (75 MHz, CDCl_3_) δ (ppm): 148.92, 146.07, 137.61, 133.88, 130.64, 126.33, 123.06, 122.31, 56.31, 37.17, 33.31, and 23.21. ^19^F NMR (565 MHz, CDCl_3_): singlet at δ −81.69 ppm (Tf_2_N-CF_3_). ESI-MS (positive mode): 215.2 *m*/*z* [M − Tf_2_N^−^, C_14_H_19_N_2_]^+^.

**3-(4-(^tert^butyl)benzyl)-1-methylimidazolium bis((trifluoromethyl)sulfonyl)amide** [^tert^BBnMIm][Tf_2_N] (**3c**): Obtained in a 91% yield. ^1^H NMR (500 MHz, CDCl_3_) δ (ppm); 9.88 (1H, s, Im-H), 7.93 (1H, d, J = 2.0 Hz, Im-H), 7.74 (1H, d, J = 2.0 Hz, Im-H), 7.49–7.23 (4H, m, Ar-H), 5.39 (2H, 2, Ar-CH_2_), 3.89 (3H, s, N-CH_3_), 1.38 (9H, s, C(CH_3_)_3_). ^13^C NMR (126 MHz, CDCl_3_) δ (ppm): 149.76, 148.57, 138.64, 131.95, 129.13, 125.15, 123.88, 122.96, 55.63, 37.74, 34.34, and 31.91. ^19^F NMR (565 MHz, CDCl_3_): singlet at δ −81.65 ppm (Tf_2_N-CF_3_). ESI-MS (positive mode): 229.2 *m*/*z* [M − Tf_2_N^−^, C_15_H_21_N_2_]^+^.

**3-benzyl-1-butylimidazolium bis((trifluoromethyl)sulfonyl)amide** [BnBIm][Tf_2_N] (**3d**): Obtained in a 91% yield. ^1^H NMR (500 MHz, CDCl_3_) *δ* (ppm); 9.26 (1H, s, Im-H), 7.84 (1H, d, *J* = 1.8 Hz, Im-H), 7.78 (1H, d, *J* = 1.7 Hz, Im-H), 7.61–7.36 (5H, m, Ar-H), 5.35 (2H, 2, Ph-CH_2_), 4.19 (2H, t, *J* = 7.2 Hz, N-CH_2_CH_2_CH_2_CH_3_), 1.79 (2H, p, *J* = 7.2 Hz, N-CH_2_CH_2_CH_2_CH_3_), 1.27 (2H, m_(6)_, N-CH_2_CH_2_CH_2_CH_3_), 0.91 (3H, t, *J* = 7.3 Hz, N-CH_2_CH_2_CH_2_CH_3_). ^13^C NMR (126 MHz, CDCl_3_) δ (ppm): 149.68, 137.93, 134.63, 129.24, 128.74, 126.02, 123.14, 122.89, 55.24, 47.44, 31.60, 19.16, and 13.60. ^19^F NMR (565 MHz, CDCl_3_): singlet at δ −81.66 ppm (Tf_2_N-CF_3_). ESI-MS (positive mode): 215.2 *m*/*z* [M − Tf_2_N^−^, C_14_H_19_N_2_]^+^.

**3-(4-isopropylbenzyl)-1-butylimidazolium bis((trifluoromethyl)sulfonyl)amide** [*^iso^*PBnBIm][Tf_2_N] (**3e**): Obtained in a 85% yield. ^1^H NMR (500 MHz, CDCl_3_) *δ* (ppm); 9.91 (1H, s, Im-H), 7.92 (1H, d, *J* = 2.2 Hz, Im-H), 7.72 (1H, d, *J* = 2.1 Hz, Im-H), 7.44–7.19 (4H, m, Ar-H), 5.64 (2H, 2, Ar-CH_2_), 4.26 (2H, t, *J* = 7.4 Hz, N-CH_2_CH_2_CH_2_CH_3_), 3.28 (1H, p, *J* = 6.9 Hz, CH(CH_3_)_2_), 1.85 (2H, p, *J* = 7.2 Hz, N-CH_2_CH_2_CH_2_CH_3_), 1.33 (2H, m_(6)_, N-CH_2_CH_2_CH_2_CH_3_), 1.19 (6H, d, *J* = 7.1 Hz, CH(CH_3_)_2_) 0.91 (3H, t, *J* = 7.3 Hz, N-CH_2_CH_2_CH_2_CH_3_). ^13^C NMR (126 MHz, CDCl_3_) δ (ppm): 149.65, 146.03, 138.91, 134.53, 129.38, 128.57, 125.64, 123.58, 122.54, 55.75, 47.45, 32.38, 31.82, 23.23, 21.81, and 13.85. ^19^F NMR (565 MHz, CDCl_3_): singlet at δ −81.67 ppm (Tf_2_N-CF_3_). ESI-MS (positive mode): 257.3 *m*/*z* [M − Tf_2_N^−^, C_17_H_25_N_2_]^+^

**3-(4-(*^tert^*butyl)benzyl)-1-butylimidazolium bis((trifluoromethyl)sulfonyl)amide** [*^tert^*BBnBIm][Tf_2_N] (**3f**): Obtained in a 89% yield. ^1^H NMR (500 MHz, CDCl_3_) *δ* (ppm); 9.84 (1H, s, Im-H), 7.91 (1H, d, *J* = 2.1 Hz, Im-H), 7.74 (1H, d, *J* = 2.1 Hz, Im-H), 7.49–7.27 (4H, m, Ar-H), 5.61 (2H, 2, Ar-CH_2_), 4.28 (2H, t, *J* = 7.3 Hz, N-CH_2_CH_2_CH_2_CH_3_), 1.83 (2H, p, *J* = 7.1 Hz, N-CH_2_CH_2_CH_2_CH_3_), 1.40 (9H, s, C(CH_3_)_3_), 1.31 (2H, m_(6)_, N-CH_2_CH_2_CH_2_CH_3_), 1.19 (6H, d, *J* = 6.9 Hz, CH(CH_3_)_2_) 0.90 (3H, t, *J* = 7.2 Hz, N-CH_2_CH_2_CH_2_CH_3_). ^13^C NMR (126 MHz, CDCl_3_) δ (ppm): 149.72, 148.51, 137.92, 131.65, 129.42, 125.64, 123.63, 122.91, 55.68, 47.52, 34.41, 33.34, 31.43, 21.21, and 13.87. ^19^F NMR (565 MHz, CDCl_3_): singlet at δ −81.69 ppm (Tf_2_N-CF_3_). ESI-MS (positive mode): 271.3 *m*/*z* [M − Tf_2_N^−^, C_18_H_27_N_2_]^+^

### 3.2. Preparation of IILMEs-Mediated AgNPs

With a minor tweak, we used the optimum conditions adopted from previously reported investigations [48,49,50] to fabricate the AgNPs in situ in the microemulsions containing the BAIILs (**3a–f**). In brief, the BAIILs/TX-100/H_2_O microemulsions (IILMEs) were first prepared by mixing 0.1 g of BAIIL and 1.4 g of TX-100 in deionized water (8.5 g) for 20 min at room temperature to ensure proper blending and formation of homogeneous solutions. Afterward, a 0.1 mmol aqueous AgNO_3_ solution was added to this microemulsion and the mixture was agitated for 10 min. A diluted hydrazine hydrate solution (1 mL) was added and the reaction mixture was then stirred at 60 °C for 1 h. The solution color changes from a light yellow to a brownish yellow, which is evidence of the creation of silver nanoparticles. To ensure full reduction, a small amount of hydrazine hydrate was added. Moreover, UV-Visible spectroscopy scanning verified the production of AgNO_3_. Silver nanoparticles were recovered after being stabilized in a microemulsion system composed of AgNO_3_/BAIILs/TX-100/H_2_O by centrifuging.

### 3.3. Antimicrobial Study

Three representative ESKAPE pathogens *PA* (ATCC-27853), *KP* (ATCC-13883), and *SA* (ATCC-29737) were utlized to test the antimicrobial power of the new BAIIL-supported AgNPs. Ciprofloxacin (Cipro), the most common antibiotic used for skin bacterial treatments, was served as the “positive” control. The NODCAR in Cairo, Egypt, kindly supplied all of the bacterial species used in this study, and these bacteria were routinely cultured in nutrient broth agar (NBA). First, we inoculated Mueller–Hinton Broth (MHB) with a bacterial solution containing ~10^6^ CFU/mL and incubated the mixture at 37 °C in a 5% CO_2_ environment to establish the initial bacterial culture. We then used the Well diffusion assay (WDA) and colony forming unit (CFU) methods outlined in our previous work [51] to determine which bacterial strains were most sensitive to the novel AgNPs. The sizes of the inhibition zones (IZD, mm) were the most important factors in determining the NPs’ antibacterial effectiveness. For the CFU method, we used the following formula (Equation (1)) to determine the relative decline in bacterial colony numbers (R%):

(1) $$R\%=\frac{{\mathrm{BC}}_{CT}-{\mathrm{BC}}_{TT}}{{\mathrm{BC}}_{CT}}$$

where BC*_CT_* and BC*_TT_* are the number of bacterial colonies in growth control and treatment test tubes, respectively. The obtained results were determined using mean SEM from triplicates of each trial.

#### Minimal Inhibitory/Bactericidal Concentrations (MIC/MBC)

The antibacterial efficacy indicators, MIC and MBC, of new compounds against tested bacterial strains were determined using the microtitre broth dilution technique as described in our prior study [52]. In brief, the bacterial suspension was treated with AgNPs and antibiotics, separately, that had been pre-dispersed in DMSO and prediluted Mueller–Hinton Broth (MHB). After transferring 190 μL bacterial suspensions (10^6^ CFU/mL) to 96-well microtiter plates, AgNPs with concentrations in the range of 0.25–50.0 g/mL were added, then the plates were left to incubate at 37 °C for 24 h; controls consisted of wells that had not been treated. The Well turbidity measurements were used to calculate MIC and MBC concentrations. In order to calculate the MIC and MBC, multiple independent replicates of each sample were evaluated. The results are shown as the mean ± SEM.

### 3.4. Anti-Biofilm Study

According to our previously published work [52], the ability of the most effective antibiotics (**AgNPs-3d**, **AgNPs-3e**, and **AgNPs-3f**) to inhibit bacterial biofilm formation and eradicate the biofilms created by the tested bacterial strains (SA, PA, and KP) was studied.

### 3.5. In Vitro Cytotoxicity Study

#### 3.5.1. Cell Cultures

Healthy human skin fibroblast (HSF) cell lines were obtained from the American Type Cell Culture Collection (ATCC, Manassas, USA). Dulbecco’s Modified Eagle’s Medium (DMEM, Invitrogen/Life Technologies) was used to cultivate these cell lines, supplemented with 10% fetal bovine serum, 100 U/mL of penicillin, and 100 g/mL of streptomycin (HyClone, Thermo Scientific). The cells were maintained in a Thermo Scientific Heracell VIOS CO_2_ incubator maintained at 37 °C with 5% CO_2_ humidity.

#### 3.5.2. In Vitro Anti-Proliferative Activity

The new BAIIL-supported AgNPs were tested for their anti-breast cancer action in vitro using the MTT assay. Briefly, a 96-well plate (Falcon, NJ, USA) was used to treat cell lines (10^5^ cells/well) with a range of doses (1.56–50 µg/mL) of the tested substance, a positive control cisplatin (CDDP), and a negative control (DMSO). Sets of wells (consisting of three wells each) were assigned for each sample. Cells were incubated for 48 h at 37 °C in a 5% CO_2_ atmosphere, after which they were fixed, washed, and stained with MTT reagent, and then re-incubated for an additional 4 h. The staining media was carefully removed from the plate after incubation, and 180 μL of acidified isopropanol/well was added. The plate was then agitated at ambient temperature with a MaxQ 2000 plate shaker (Thermo Fisher Scientific Inc., MI, USA) to dissolve the formazan crystals that had formed. In order to determine the vitality of the cells, the plate was next subjected to a spectrophotometric analysis using a Stat FaxR 4200 plate reader (Awareness Technology, Inc., FL, USA).

## 4. Conclusions

This work presents the synthesis and characterization of a new class of imidazolium-supported BAIILs. (**3a–f**) by employing spectral (FTIR, NMR, and ESI-MS), thermal, and viscosity techniques. BAIILs were used as media and stabilizing agents for hydrazine-hydrate-catalyzed AgNO_3_ reduction in BAIILs/TX-100/H_2_O microemulsions into extremely small AgNPs. Unlike more traditional approaches, this synthetic process permits the synthesis of AgNPs networks with narrower PSD. Interestingly, AgNPs isolated from the BAIIL bearing the bulkiest substituents (*tert*-butyl and *n*-butyl) (**3f**) displayed almost no NPs agglomeration. This study demonstrates that a simple step can be taken to achieve well-separated AgNPs—the addition of an electron-donating bulky para-substituent on the phenyl ring of the benzylimidazolium cation. All of the AgNPs-BAIILs tested in the in vitro antibacterial assay against ESKAPE pathogens showed very strong antibiotic properties, as evidenced by their DIZ and MIC/MBC values. Additionally, Gram-negative bacterial strains were more treatment-resistant than Gram-positive ones. Human skin fibroblast (HSF) cell lines were employed in an MTT experiment to measure the growth inhibitory effects of new drugs. The MTT cytotoxicity assay showed that the novel AgNPs had no great effect on the HSF cells. Consequently, the BAIIL-coated AgNPs could offer safe and promising antibiotic candidates for skin bacterial infection treatments. 

## Figures and Tables

**Table 1:** Physicochemical characteristics of new BAIILs.

TAAI.	MW (g/mol)	Appearance	LogS *^a^*	CLogP *^b^*	D (g/cm^3^) *^c^*	*η* (cP) *^d^*	T_dec_ (°C) *^e^*
**3a**	453.37	Yellow oil	−5.719	−0.352	1.468	421.15	407
**3b**	495.46	Orange oil	−6.820	−0.262	1.397	426.85	402
**3c**	509.48	Yellow oil	−7.150	0.137	1.551.	439.25	412
**3d**	495.46	Orange oil	−6.735	−0.102	1.298	512.67	397
**3e**	537.54	Brown oil	−7.834	1.325	1.213	521.83	394
**3f**	551.56	Orange oil	−8.163	1.724	1.311	543.25	401

^a,b^ calculated using ChemDraw 16; ^c^ density measured using COSMOtherm at 50 °C; ^d^ viscosity measured at 25 °C using a capillary viscometer; ^e^ decomposition temperature from DTG curves.

**Table 2:** MIC and MBC values (μg/mL) of new BAIILs-supported AgNPs against ESKAPE pathogens, as compared to clinical antibiotics (GM and TC).

Sample	Size (nm)	*SA*		*PA*		*KB*	
		MIC ± SD	MBC ± SD	MIC ± SD	MBC ± SD	MIC ± SD	MBC ± SD
**AgNPs-3a**	2.9	3.25 ± 0.25	3.75 ± 0.31	8.76 ± 0.32	8.85 ± 0.35	9.32 ± 0.34	9.50 ± 0.37
**AgNPs-3b**	2.4	2.25 ± 0.12	2.25 ± 0.15	7.07 ± 0.19	7.15 ± 0.45	8.87 ± 0.11	8.05 ± 0.25
**AgNPs-3c**	2.2	1.95 ± 0.15	2.07 ± 0.19	5.85 ± 0.25	5.95 ± 0.33	7.35 ± 0.37	7.48 ± 0.33
**AgNPs-3d**	2.0	1.76 ± 0.15	1.95 ± 0.11	5.55 ± 0.29	5.65 ± 0.37	7.15 ± 0.21	7.23 ± 0.25
**AgNPs-3e**	1.7	0.85 ± 0.11	0.95 ± 0.23	2.75 ± 0.36	2.85 ± 0.45	4.45 ± 0.29	4.55 ± 0.41
**AgNPs-3f**	1.5	0.25 ± 0.12	0.35 ± 0.18	1.36 ± 0.27	1.39 ± 0.28	2.22 ± 0.15	2.25 ± 0.19
**Cipro**	-	5.20 ± 0.16	5.75 ± 0.25	7.75 ± 0.23	8.05 ± 0.31	10.13 ± 0.56	10.55 ± 0.48

NA = not assigned.

## Data Availability

Not applicable.

## Supplementary Materials

The following supporting information can be downloaded at: https://www.mdpi.com/article/10.3390/antibiotics12020247/s1, Figure S1: TG curves of TBAIILs. Figure S2: Photographs of inhibition zones for the most active antibiotics (**AgNPs-3e** and **AgNPs-3f**). Table S1: MIC and MBC values (μg/mL) of new BAIILs-supported AgNPs against different pathogens, in comparison to previously reported ones.
