# Supplementary material for: New Ionic Liquid Microemulsion-Mediated Synthesis of Silver Nanoparticles for Skin Bacterial Infection Treatments
Source: Antibiotics (Basel). 2023 Jan 25;12(2):247. doi: 10.3390/antibiotics12020247 (PMC9952689; doi:10.3390/antibiotics12020247)
Supplement: Supplementary file 1 [file antibiotics-12-00247-s001.zip › antibiotics-2140142-supplementary.pdf]

New Ionic Liquid Microemulsion-Mediated Synthesis of Silver Nanoparticles for Skin Bacterial Infection Treatments

Fayez Althobaiti [1], Ola A. Abu Ali [2], Islam Kamal [3], Mohammad Y. Alfaifi [4], Ali A Shati [4], Eman Fayad [1], Serag Eldin I. Elbehairi [3,5], Reda F. M. Elshaarawy [6,7,*] and W. Abd El-Fattah [8,9,*]

[1] Department of Biotechnology, Faculty of Sciences, Taif University, P.O. Box 11099, Taif 21944, Saudi Arabia
[2] Department of Chemistry, College of Science, Taif University, P.O.Box 11099 Taif 21944, Saudi Arabia
[3] Department of Pharmaceutics, Faculty of Pharmacy, Port Said University, 42526 Port Said, Egypt
[4] Biology Department, Faculty of Science, King Khalid University, 9004 Abha, Saudi Arabia
[5] Cell Culture Lab, Egyptian Organization for Biological Products and Vaccines (VACSERA Holding Company), 51 Wezaret El-Zeraa St., Agouza, Giza, Egypt
[6] Department of Chemistry, Faculty of Science, Suez University, 43533 Suez, Egypt
[7] Institut für Anorganische Chemie und Strukturchemie, Heinrich-Heine Universität Düsseldorf, Düsseldorf, Germany
[8] Chemistry Department, College of Science, IMSIU (Imam Mohammad Ibn Saud Islamic University), P.O. Box 5701, Riyadh 11432, Saudi Arabia
[9] Department of Chemistry, Faculty of Science, Port Said University, Port Said, Egypt
* Correspondence: reda.elshaarawy@suezuniv.edu.eg (R.F.M.E.), Tel. +201017377216 (R.F.M.E.); wabdulfatah@imamu.edu.sa (W.A.).

1. Materials and Instrumentation

Chemicals were obtained from the following suppliers and used without further purification: toluene, cumene, tert-butylbenzene, 1-methylimidazole, 1-n-butyllimidazole lithium bis(trifluoromethanesulfonimide), and anhydrous $MgCl_2$ (Sigma–Aldrich); Zinc iodide (Acros).

Melting points were *measured* using a BÜCHI Melting point B-540 apparatus; all *melting points were measured* in *open glass capillaries* and are uncorrected. Elemental analyses for C, H, N, were performed with a Perkin–Elmer 263 elemental analyzer. FT-IR spectra were recorded on a BRUKER Tensor-37 FT-IR spectrophotometer in the range 400–4000 cm^{-1} as KBr disc in the 4000-550 cm^{-1} region with 2 cm^{-1} resolution or with an ATR (attenuated total reflection) unit (Platinum ATR-QL, diamond). For signal intensities the following abbreviations were used: br (broad), sh (sharp), w (weak), m (medium), s (strong), vs (very strong). UV/Vis spectra were measured at 25 °C in ethanol (10^{-5} mol/L) on a Shimadzu UV-2450 spectrophotometer using quartz cuvettes (1 cm). NMR-spectra were obtained with a Bruker Avance DRX200 (200 MHz for ^{1}H) or Bruker Avance DRX500 (125 and 470 MHz for ^{13}C and ^{19}F respectively) spectrometer with calibration to the residual proton solvent signal in DMSO-d_6 (^{1}H NMR: 2.52 ppm, ^{13}C NMR: 39.5 ppm), $CDCl_3$ (^{1}H NMR: 7.26 ppm, ^{13}C NMR: 77.16 ppm) against TMS (δ = 0.00 ppm) for ^{1}H and ^{13}C, 85% phosphoric acid (δ = 0.00 ppm) for ^{31}P and $CFCl_3$ (δ = 0.00 ppm) for ^{19}F NMR. Multiplicities of the signals were specified s (singlet), d (doublet), t (triplet), q (quartet) or m (multiplet). The ESI-MS of the synthesized compounds were acquired in the linear mode for positive ions on a UHR-QTOF maXis 4G (Bruker Daltonics) and BRUKER Ultraflex MALDI-TOF instrument equipped with a 337 nm nitrogen laser pulsing at a repetition rate of 10 Hz. The 2+ charge assignment of ions in HR-ESI-MS was confirmed by the m/z = 0.5 difference between the isotope peaks (x, x+1, x+2). Peaks with chlorine showed the isotope ratio $^{35/37}$Cl = 75.8:24.2.

2. Synthesis of 1-alkyl-4-(chloromethyl)benzene (1a-c)

General procedure: A flask was charged with 5 mol% of ZnI_2 (1.3 mmol), chlorosulfonicacid (31 mmol) and CH_2Cl_2 (30 mL), followed by dropwise addition of dimethoxymethane (31 mmol) at -10 °C. After stirring the reaction mixture at -10°C for 30min, the aromatic compound (26 mmol) was slowly added.The resulting mixture was then stirred at 5-10 °C for the time 0.5 - 2 h. The reaction was monitored by TLC analysis.

After completion, the reaction was quenchedby addition of water (10 mL) in an ice bath. After extractionwith CH_2Cl_2 (3 x 20 mL), the organic phase was washedwith 5% sodium carbonate solution (2 x 10 mL), water (2 x10 mL) and brine (2 x 20 mL), then evaporated to drynessunder reduced pressure. The residue was purified by flashcolumn chromatography on a silica gel using petroleumether (boiling range: 60-90°C) and ethyl acetate as eluents to give the desired product.

1-(Chloromethyl)-4-isopropylbenzene (**1b**) [24]: Colorless liquid. ^{1}H NMR (500 MHz, $CDCl_3$) δ (ppm): 7.41 (d, *J* = 8.1 Hz, 2H), 7.32 (d, *J* = 8.1Hz, 2H), 4.66 (s, 2H), 3.05–2.93 (m, 1H), 1.35 (d, *J* = 7.4 Hz, 6H); 13C NMR (125 MHz, $CDCl_3$) δ (ppm): 146.3, 138.7, 128.6, 126.7, 46.0, 33.9, 23.9.

*1-(*tert*-Butyl)-4-(chloromethyl)benzene* (**1c**) [24]: Colorless liquid. ^{1}H NMR (500 MHz, $CDCl_3$) δ (ppm): δH 7.46 (d, *J* = 8.4 Hz, 2H), 7.42–7.39 (m, 2H), 4.64 (s, 2H), 1.40 (s, 9H); 13C NMR (125 MHz, CDCl3) δ (ppm):151.4, 134.5, 128.3, 125.6, 46.0, 34.5, 31.3.

3. Synthesis of benzyl alkyl imidazolium chloride [$R^1BnImR^2]^+Cl^–$ ionic liquids (2a-f)

Into a three-neck round-bottom flask, benzyl chloride derivatives (0.03 mol) were added dropwise to a solution of 1-alkylimidazole (0.03 mol) in acetonitrile (ACN) (30 mL) at room temperature under stirring. The mixture was then heated under reflux in a N_2 atmosphere for about 48 h, until no starting material was detected by thin layer chromatography (TLC). After the reaction was completed, ACN was evaporated into vacuo, and the yellowish oily product was washed three times with refluxing ethyl acetate (25 mL) at 353 K to eliminate any residual unreacted starting materials. The pure oily products were then dried for 48 h in a vacuum oven (40 mbar) at 333 K. Samples of the produced TBAILs (**2a-f**) were chracterized as follow;

3-benzyl-1-methyl-1H-imidazol-3-ium chloride [BnMIm]Cl (**2a**): Obtained in a 93% yield. ^{1}H NMR (500 MHz, $CDCl_3$) *δ* (ppm); 9.39 (1H, s, Im-H), 7.89 (1H, d, *J* = 1.9 Hz, Im-H), 7.75 (1H, d, *J* = 1.9 Hz, Im-H), 7.69–7.45 (5H, m, Ar-H), 5.52 (2H, 2, Ph–<u>CH_2</u>), 3.84 (3H, s, N–<u>CH_3</u>). ESI-MS (positive mode): 173.2 m/z [M – $Cl^–$, $C_{11}H_{13}N_2]^+$

3-(4-isopropylbenzyl)-1-methyl-1H-imidazol-3-ium chloride [^{iso}PBnMIm]Cl (**2b**): Obtained in a 90% yield. ^{1}H NMR (500 MHz, $CDCl_3$) *δ* (ppm); 9.48 (1H, s, Im-H), 7.91 (1H, d, *J* = 1.8 Hz, Im-H), 7.71 (1H, d, *J* = 1.8 Hz, Im-H), 7.47–7.30 (4H, m, Ar-H), 5.57 (2H, 2, Ar–<u>CH_2</u>), 3.98 (3H, s, N–<u>CH_3</u>), 3.32 (1H, p, *J* = 1.9 Hz, <u>CH</u>($CH_3)_2$), 1.23 (6H, d, *J* = 7.0 Hz, CH(<u>$CH_3)_2$</u>). ESI-MS (positive mode): 215.2 m/z [M – $Cl^–$, $C_{14}H_{19}N_2]^+$

3-(4-(tert-butyl)benzyl)-1-methyl-1H-imidazol-3-ium chloride [^{tert}BBnMIm]Cl (**2c**): Obtained in a 92% yield. ^{1}H NMR (500 MHz, $CDCl_3$) *δ* (ppm); 9.83 (1H, s, Im-H), 7.89 (1H, d, *J* = 1.8 Hz, Im-H), 7.72 (1H, d, *J* = 1.8 Hz, Im-H), 7.51–7.26 (4H, m, Ar-H), 5.58 (2H, 2, Ar–<u>CH_2</u>), 4.01 (3H, s, N–<u>CH_3</u>), 1.40 (9H, s, C(<u>$CH_3)_3$</u>). ESI-MS (positive mode): 229.2 m/z [M – $Cl^–$, $C_{15}H_{21}N_2]^+$

3-benzyl-1-butyl-1H-imidazol-3-ium chloride [BnBIm]Cl (**2d**): Obtained in a 89% yield. ^{1}H NMR (500 MHz, $CDCl_3$) *δ* (ppm); 9.39 (1H, s, Im-H), 7.89 (1H, d, *J* = 1.9 Hz, Im-H), 7.75 (1H, d, *J* = 1.9 Hz, Im-H), 7.69–7.45 (5H, m, Ar-H), 5.52 (2H, 2, Ph–<u>CH_2</u>), 3.84 (3H, s, N–<u>CH_3</u>). ESI-MS (positive mode): 215.2 m/z [M – $Cl^–$, $C_{14}H_{19}N_2]^+$

3-(4-isopropylbenzyl)-1-butyl-1H-imidazol-3-ium chloride [^{iso}PBnBIm]Cl (**2e**): Obtained in a 83% yield. ^{1}H NMR (500 MHz, $CDCl_3$) *δ* (ppm); 9.48 (1H, s, Im-H), 7.91 (1H, d, *J* = 1.8 Hz, Im-H), 7.71 (1H, d, *J* = 1.8 Hz, Im-H), 7.47–7.30 (4H, m, Ar-H), 5.57 (2H, 2, Ar–<u>CH_2</u>), 3.98 (3H, s, N–<u>CH_3</u>), 3.32 (1H, p, *J* = 6.9 Hz, <u>CH</u>($CH_3)_2$), 1.23 (6H, d, *J* = 7.0 Hz, CH(<u>$CH_3)_2$</u>). ESI-MS (positive mode): 257.3 m/z [M – $Cl^–$, $C_{17}H_{25}N_2]^+$

3-(4-(tert-butyl)benzyl)-1-butyl-1H-imidazol-3-ium chloride [^{tert}BBnBIm]Cl (**2f**): Obtained in a 87% yield. ^{1}H NMR (500 MHz, $CDCl_3$) *δ* (ppm); 9.83 (1H, s, Im-H), 7.89 (1H, d, *J* = 2.0 Hz, Im-H), 7.72 (1H, d, *J* = 2.0 Hz, Im-H), 7.51–7.26 (4H, m, Ar-H), 5.58 (2H, 2, Ar–<u>CH_2</u>), 4.01 (3H, s, N–<u>CH_3</u>), 1.40 (9H, s, C(<u>$CH_3)_3$</u>). ESI-MS (positive mode): 271.3 m/z [M – $Cl^–$, $C_{18}H_{27}N_2]^+$

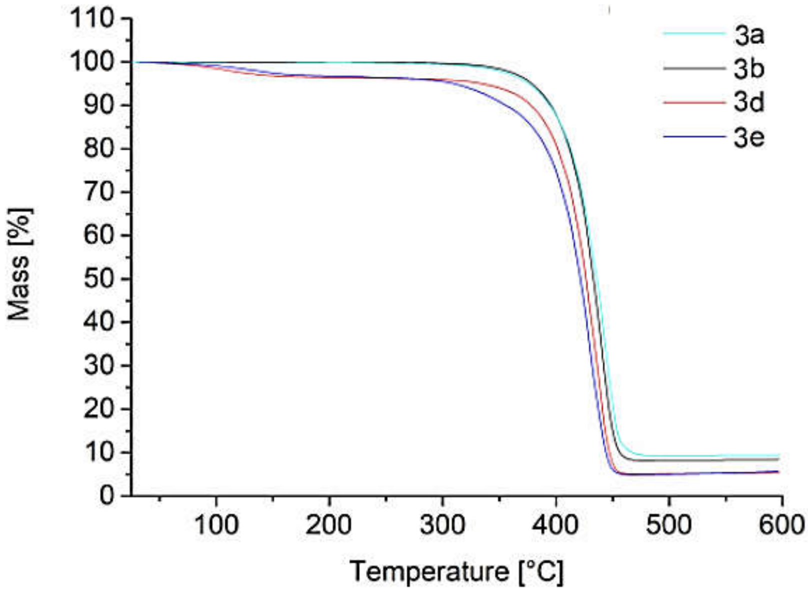

Figure S1. TG curves of TBAIILs.

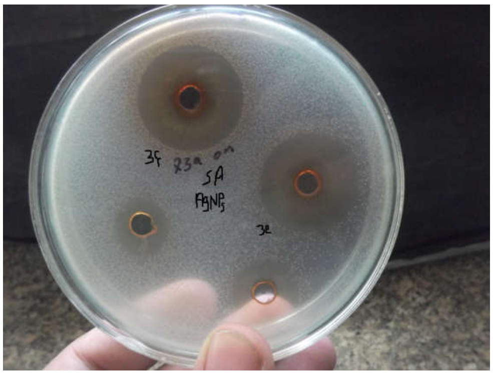

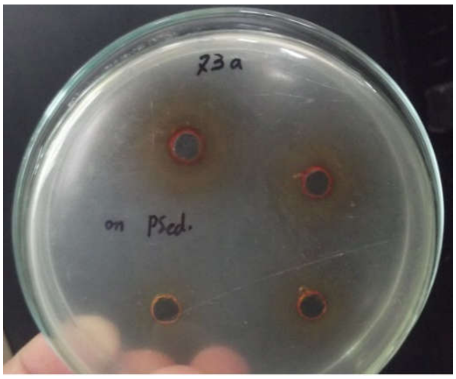

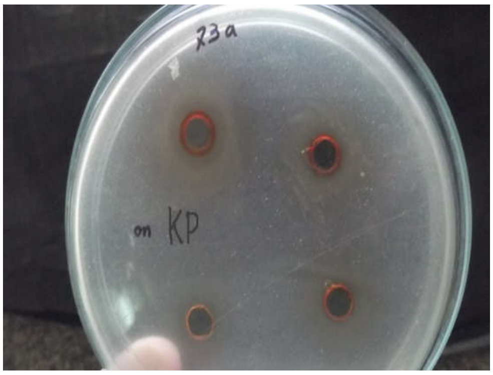

Figure S2. Photographs of inhibition zones for the most active antibiotics (**AgNPs-3e** and **AgNPs-3f**).

Table S1. MIC and MBC values (µg/mL) of new BAIILs-supported AgNPs against different pathogens, in comparison to previously reported ones.

Sample	Size (nm)	*SA*		*PA*		*KB*		Ref.
		**MIC** ± SD	**MBC** ± SD	**MIC** ± SD	**MBC** ± SD	**MIC** ± SD	**MBC** ± SD	
AgNPs-3a	2.9	3.25 ±0.25	3.75 ±0.31	8.76 ±0.32	8.85 ±0.35	9.32 ±0.34	9.50 ±0.37	This work
AgNPs-3b	2.4	2.25 ±0.12	2.25 ±0.15	7.07 ±0.19	7.15 ±0.45	8.87 ±0.11	8.05 ±0.25	This work
AgNPs-3c	2.2	1.95 ±0.15	2.07 ±0.19	5.85 ±0.25	5.95 ±0.33	7.35 ±0.37	7.48 ±0.33	This work
AgNPs-3d	2.0	1.76 ±0.15	1.95 ±0.11	5.55 ±0.29	5.65 ±0.37	7.15 ±0.21	7.23 ±0.25	This work
AgNPs-3e	1.7	0.85 ±0.11	0.95 ±0.23	2.75 ±0.36	2.85 ±0.45	4.45 ±0.29	4.55 ±0.41	This work
AgNPs-3f	1.5	0.25 ±0.12	0.35 ±0.18	1.36 ±0.27	1.39 ±0.28	2.22 ±0.15	`2.25 ±0.19	This work
AgNPs-$C_{12}Im^{a}$	9	16	16	32	32	NA	NA	[45]
AgNPs-$C_{18}Im^{b}$	8.6	256	256	256	256	NA	NA	[45]
AgNPs-$C_{12}Py^{c}$	18.49	16	16	64	64	NA	NA	[45]
AgNPs-$C_{18}Py^{d}$	6.71	128	128	128	128	NA	NA	[45]
AgNPs[BMIM][BF_4]	132	125	125	NA	NA	NA	NA	[46]
AgNPs[EMIM][BF_4]	360	500	500	NA	NA	NA	NA	[46]

NA= not assigned. [a]1-dodecyl-3-methylimidazolium chloride; [b] 1-octadecyl-3-methylimidazolium chloride; [c] 1-dodecyl pyridinium chloride; [d] 1-octadecyl pyridinium chloride; [BMIM][BF_4]1-butyl-3-methylimidazolium tetrafluoroborate ; [EMIM][BF_4]).1-ethyl-3-methylimidazolium tetrafluoroborate.
